# Case Report: Successful treatment of a novel variant of *CARD14*-mutated juvenile Pityriasis rubra pilaris with ixekizumab

**DOI:** 10.3389/fmed.2025.1637045

**Published:** 2025-07-22

**Authors:** Laura Millak, Matthias Hahn, Judith Fischer, Sebastian Volc

**Affiliations:** ^1^Department of Dermatology, University Hospital Tuebingen, Tuebingen, Germany; ^2^Institute of Human Genetics, Medical Center, University of Freiburg, Freiburg, Germany

**Keywords:** Pityriasis rubra pilaris, case report, ixekizumab, *CARD14*, pediatric dermatology

## Abstract

Pityriasis rubra pilaris is a rare inflammatory papulosquamous skin disease without any approved treatment options. Variants in the *CARD14* (caspase recruitment domain family member 14) gene have been identified to play a role in the pathophysiology of atypical juvenile PRP by activating the IL-23/IL-17A cytokine axis, highlighting this pathway as a potential target of therapy. Here, we present a case of successful treatment with ixekizumab, a humanized monoclonal anti-IL-17A antibody, in an atypical juvenile PRP (type V) patient with a novel variant of *CARD14* mutation.

## 1 Introduction

Pityriasis rubra pilaris is a rare inflammatory disease characterized by red-orange scaling plaques with classic “islands of sparing” of unaffected skin, keratotic follicular papules, and palmoplantar keratoderma. PRP can be divided into six clinical subtypes affecting pediatric and adult patients (1). *CARD14* (caspase recruitment domain family member 14) gene variants have been found to be involved in the pathophysiology of atypical juvenile (type V) PRP leading to activation of the IL-23/IL-17A cytokine axis (2–4). Ixekizumab is a humanized monoclonal antibody targeting IL-17A. Here, we present a case of an atypical juvenile PRP (type V) patient with a novel variant of *CARD14* mutation successfully treated with ixekizumab.

## 2 Case description

**FIGURE 1:**
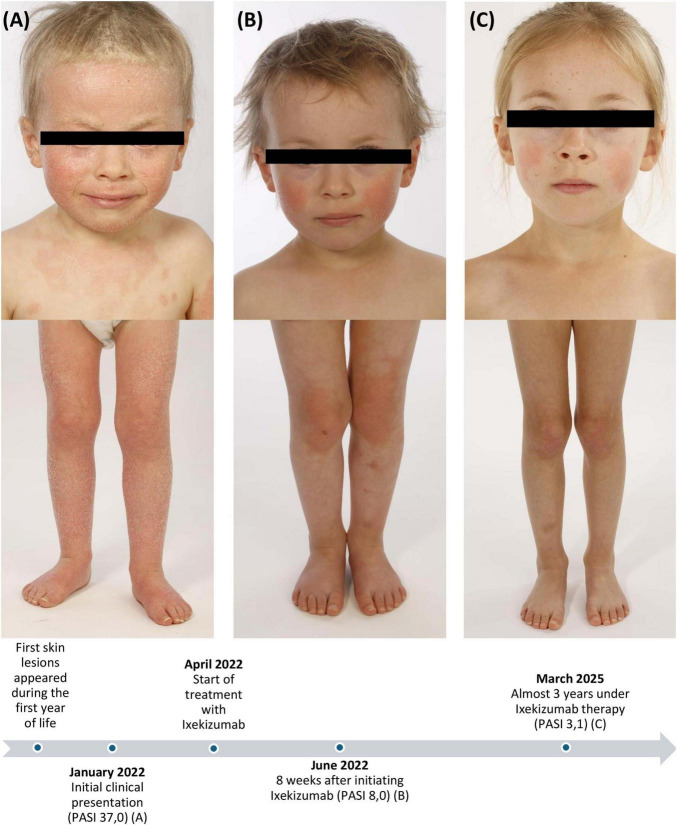
Clinical images and case timeline: Initial clinical presentation with generalized well-demarcated confluent scaly orange-red plaques, thick scaly plaques on the scalp and bilateral ectropion before treatment **(A)**, 8 weeks after initiating ixekizumab **(B)**, and almost 3 years under ixekizumab therapy **(C)**. PASI, Psoriasis Area and Severity Index.

A 3-years-old caucasian girl presented to our dermatology department at the University Hospital Tuebingen with generalized well-demarcated confluent scaly orange-red plaques with distinct “islands of sparing” of unaffected skin. The further physical examination revealed prominent facial involvement of cheeks and ears with erythematosquamous plaques, palmar and plantar waxy orange-red keratoderma, bilateral ectropion and extensive thick scaly plaques on the scalp (Figure 1A).

According to the family, initial skin lesions appeared during the first year of life. Prior to the patient’s presentation at our department, the patient had been diagnosed with a

mixed form of psoriasiform dermatitis and atopic eczema. She had been treated with topical steroids, but without sufficient response. Her growth and development were regular, with no signs suggesting a syndromic disorder. Family history was negative for psoriasis or other erythrosquamous skin diseases.

**FIGURE 2:**
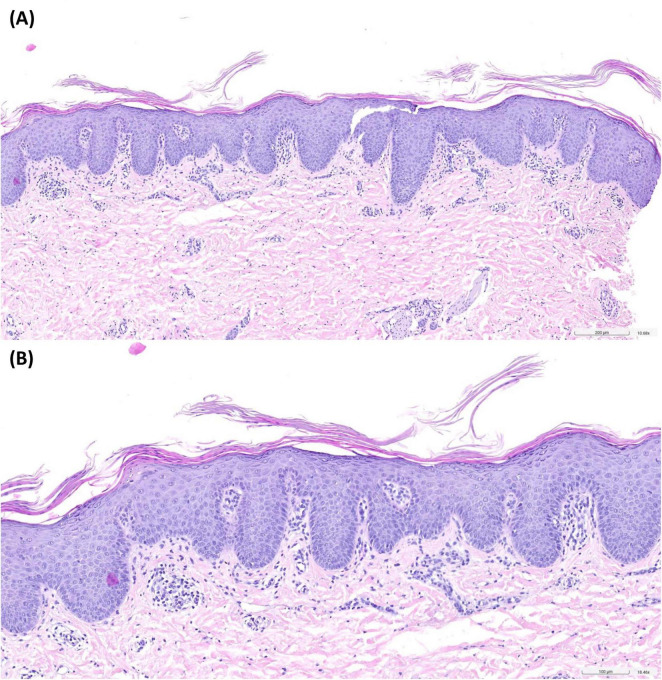
Histopathology of an affected area of the back revealed regular but plump acanthosis of the epidermis with checkerboard-like alternating orthokeratosis and parakeratosis. Sparse dermal perivascular lymphohistiocytic infiltrates. Hematoxylin-eosin-staining, 10-fold **(A)** and 18-fold **(B)** magnification.

We took a skin biopsy from an affected area of the back. Histology showed regular but plump acanthosis of the epidermis with checkerboard-like alternating orthokeratosis and parakeratosis and sparse dermal perivascular lymphohistiocytic infiltrates (Figure 2).

Based on clinical and histopathological findings, the diagnosis of PRP was made. To validate this, we performed a genetic analysis of *CARD14*, identifying the heterozygous variant c.373A>C, p.(Thr125Pro) in exon 4. This variant has not yet been described in databases such as gnomAD or HGMD, and is currently classified as a variant of unknown significance (VUS).

Due to the severity and lack of response in spite of intensive topical therapy, we opted for targeted systemic therapy. Ixekizumab is a humanized monoclonal anti-IL-17A antibody with EMA approval for treatment of moderate-to-severe plaque psoriasis in children from the age of 6 years. Ixekizumab was chosen due to the approval for psoriasis in children with beneficial safety and positive data for treatment of PRP in adults (5). Due to the absence of approved therapeutic options for PRP, the use of ixekizumab was off-label. The treatment was weight-adjusted, started with a 40 mg loading dose, followed by 20 mg every 4 weeks. Monitoring was carried out in accordance with the in-label treatment protocol for psoriasis. Given the absence of a validated severity score of PRP, the Psoriasis Area and Severity Index (PASI) was used due to the clinical overlap with psoriasis, particulary the shared presentation of erythematosquamous plaques on the extensor surfaces. A noticeable clinical response was observed in the first follow-up after 8 weeks with reduction in PASI from 37,0 to 8,0 and declining degree of ectropion (Figure 1B). According to the parents, the quality of life had been clearly improved. Almost 3 years later, we still observe a good response with residual lesions on the cheeks and elbows (PASI 3,1) (Figure 1C). So far, no side effects have been observed. Blood testing was conducted every 6 months with no relevant abnormalities. Although PRP can be self-limiting in contrast to psoriasis, we plan to continue the therapy with ixekizumab, as the patient still presents residual lesions.

## 3 Discussion

Pityriasis rubra pilaris has been classified into 6 subtypes, which are differentiated by clinical features, age at onset, and disease duration. These subtypes are classical adult-onset (Type I); atypical adult-onset (Type II); classical juvenile-onset (Type III); circumscribed juvenile-onset (Type IV); atypical juvenile-onset (Type V); and HIV-associated (Type VI) (1, 6, 7). Atypical juvenile (Type V) PRP is most commonly associated with familial forms of PRP and variants of the *CARD14* gene (8). While the novel variant p.(Thr125Pro) can currently only be classified as VUS, pathogenic missense variants in the neighboring region between amino acids 124 and 127 have already been identified in patients with PRP.

Additionally, *CARD14* variants also have been found to be associated with forms of psoriasis, including (generalized) pustular psoriasis, suggesting that these conditions share pathophysiological mechanisms with PRP (9, 10). *CARD14* variants have been identified to be a predisposing factor for autoinflammatory keratinization. These gain-of-function variants enhance nuclear factor κB (NF-κB) activation in keratinocytes, resulting in recruitment and differentiation of inflammatory cells with increased production of IL-17 and IL-22 by T cells and IL-23 by dendritic cells (11). However, in PRP patients without *CARD14* mutations, NF-κB is also activated through IL-1ß signaling, resulting in upregulation of CCL20 expression and subsequent activation of TH17 cells (12). It remains unknown whether specific *CARD14* variants are associated with particular phenotypes, because wide ranging Genome-Wide Association Study (GWAS) are lacking.

Due to the shared pathophysiological mechanisms, PRP and psoriasis present similar morphological features, characterized by well-demarcated erythematosquamous plaques predominantly affecting the extensor surfaces. In 2018, the term *CARD14*-associated papulosquamous eruption (CAPE) was proposed as a separate entity to describe a spectrum of patients with clinical characteristics of psoriasis and PRP (13). CAPE is characterized by an early age of onset, prominent facial involvement, and insufficient response to conventional therapies. In our patient, some clinical characteristics of CAPE are also met, such as manifestation within the first year of life and prominent facial involvement.

As there are significant histopathological similarities between PRP and CAPE, clinical classification of juvenile patients with PRP might be challenging (14). Many patients are diagnosed as type V, which is sometimes used as a general category for all juvenile and familial cases of PRP. Therefore, we would like to emphasize the importance of a genetic testing for *CARD14* mutations in patients with familiar and early-onset PRP. It is necessary to establish clear and standardized diagnostic criteria for CAPE and type V PRP to ensure unambiguous and accurate classification.

Since PRP, CAPE and psoriasis share common pathophysiological mechanisms, targeting the IL-23/IL-17 signaling pathway offers a promising strategy for therapeutic intervention (3). Currently, ustekinumab, a combined IL-12 and IL-23 inhibitor, holds the most evidence for the therapy of PRP with *CARD14* gene variations or CAPE (15, 16). However, targeted inhibition of IL-17A with secukinumab also showed promising results (17, 18). A single-arm, investigator-initiated trial treating 12 adult patients with moderate to severe PRP, including one with juvenile-onset, with ixekizumab showed improvement in their skin condition and improvement of quality of life (5). Furthermore, several case reports of successful treatment with ixekizumab in adult patients with CAPE have been published (19, 20).

In children with plaque psoriasis, ixekizumab has demonstrated long-term effectiveness and a favorable safety profile, which led to approval from the age of 6 (21). As the diagnosis PRP is rarely made early in children, it is difficult to carry out a prospective clinical study. Similar to the reported improvement in adults, a case report of a 6-years-old boy with juvenile PRP showed rapid response after starting treatment with ixekizumab (22). However, it must be emphasized that the *CARD14* gene status was unknown.

It is uncertain if therapeutic responses to different biological molecules can be predicted based on *CARD14* variations. A study involving 19 patients with PRP, of whom 10 carried *CARD14* variants, found no correlation between genetic background and treatment success (23). Due to the small numbers of participants, further investigations are necessary.

To our knowledge, this is the first case of a pediatric patient with a *CARD14*-mutated and histological confirmed PRP successfully treated for 3 years with ixekizumab. Additionally, we found a novel *CARD14* gene variant in this patient. Despite promising case reports, therapy guidelines and long-term data of children with PRP treated with modern targeted therapies are needed.

## Data Availability

The original contributions presented in this study are included in this article/supplementary material, further inquiries can be directed to the corresponding author.
